# Synergistic Reinforcement with SEBS-g-MAH for Enhanced Thermal Stability and Processability in GO/rGO-Filled PC/ABS Composites

**DOI:** 10.3390/polym16182554

**Published:** 2024-09-10

**Authors:** Fatin Najwa Joynal Abedin, Ahmad Noor Syimir Fizal, Abbas F. M. Alkarkhi, Nor Afifah Khalil, Ahmad Naim Ahmad Yahaya, Md. Sohrab Hossain, Sairul Izwan Safie, Nurul Ain Ismail, Muzafar Zulkifli

**Affiliations:** 1 Malaysian Institute of Chemical and Bioengineering Technology, University Kuala Lumpur, Alor Gajah 78000, Melaka, Malaysia; fatin.joynal28@s.unikl.edu.my (F.N.J.A.); nafifah.khalil@s.unikl.edu.my (N.A.K.); ahmadnaim@unikl.edu.my (A.N.A.Y.); 2 Centre for Sustainability of Mineral and Resource Recovery Technology (SMaRRT) (Pusat ALAM), Universiti Malaysia Pahang, Lebuh Persiaran Tun Khalil Yaakob, Gambang 26300, Pahang, Malaysia; syimirahmad@gmail.com (A.N.S.F.); nurulainis@umpsa.edu.my (N.A.I.); 3 Universiti Kuala Lumpur Business School, Kampung Datuk Keramat, Kuala Lumpur 54000, Wilayah Persekutuan Kuala Lumpur, Malaysia; alkarkhii@gmail.com; 4 HICoE-Centre for Biofuel and Biochemical Research, Institute of Sustainable Energy and Resources, Department of Fundamental and Applied Sciences, Universiti Teknologi PETRONAS, Seri Iskandar 32610, Perak Darul Ridzuan, Malaysia; sohrab.hossain@utp.edu.my; 5 Plant Engineering Technology Section, UniKL Malaysian Institute of Industrial Technology, Jalan Persiaran Ilmu, Bandar Seri Alam, Johor Bahru 81750, Johor, Malaysia; sairulizwan@unikl.edu.my; 6 Green Chemistry and Sustainability Cluster, Branch Campus, Malaysian Institute of Chemical and Bioengineering Technology, University Kuala Lumpur, Alor Gajah 78000, Melaka, Malaysia

**Keywords:** compatibilisation, synergistic reinforcement, toughening mechanism, thermally stable, graphene derivatives, polycarbonate, acrylonitrile butadiene styrene, polymer nanocomposites

## Abstract

The integration of compatibilisers with thermoplastics has revolutionised the field of polymer composites, enhancing their mechanical, thermal, and rheological properties. This study investigates the synergistic effects of incorporating SEBS-g-MAH on the mechanical, thermal, and rheological properties of polycarbonate/acrylonitrile-butadiene-styrene/graphene oxide (PC/ABS/GO) (PAGO) and the properties of polycarbonate/acrylonitrile-butadiene-styrene/graphene oxide (PC/ABS/rGO) (PArGO) composites through the melt blending method. The synergistic effects on thermal stability and processability were analysed by using thermogravimetry (TGA), melt flow index (MFI), and Fourier-transform infrared spectroscopy (FTIR). The addition of SEBS-g-MAH improved the elongation at break (EB) of PAGO and PArGO up to 33% and 73%, respectively, compared to the uncompatibilised composites. The impact strength of PAGO was synergistically enhanced by 75% with the incorporation of 5 phr SEBS-g-MAH. A thermal analysis revealed that SEBS-g-MAH improved the thermal stability of the composites, with an increase in the degradation temperature (T_80%_) of up to 17% for PAGO at 1 phr SEBS-g-MAH loading. The compatibilising effect of SEBS-g-MAH was confirmed by FTIR analysis, which indicated interactions between the maleic anhydride groups and the PC/ABS matrix and GO/rGO fillers. The rheological measurements showed that the incorporation of SEBS-g-MAH enhanced the melt flowability (MFI) of the composites, with a maximum increase of 38% observed for PC/ABS. These results demonstrate the potential of SEBS-g-MAH as a compatibiliser for improving the unnotched impact strength (mechanical), thermal, and rheological properties of PC/ABS/GO and PC/ABS/rGO composites, achieving a synergistic effect.

## 1. Introduction

Advanced polymer composites with enhanced mechanical, thermal, and rheological properties are a significant area of research in materials science. Polycarbonate/acrylonitrile-butadiene-styrene (PC/ABS) blends are commonly used for various industrial applications due to their excellent toughness and processability. PC/ABS blends often encounter limitations from the immiscibility of the constituent polymers [1,2,3,4,5]. The lack of compatibility between PC and ABS leads to poor interfacial bonding, resulting in compromised mechanical properties and reduced quality of the final products.

Incorporating fillers such as graphene oxide (GO) and reduced graphene oxide (rGO) shows a promising strategy to improve these properties significantly. While the exact structure of GO remains elusive, its potential applications span a wide range of fields, including energy storage, electronics, biomedicine, and environmental remediation [6,7,8,9,10,11]. In energy storage applications, GO is a promising material for supercapacitors and batteries due to its large surface area and high electrical conductivity [12]. In electronics, GO finds applications in transparent electrodes, flexible devices, and conductive composites. GO’s unique properties in biomedicine make it a valuable material for drug delivery, tissue engineering, and biosensing [12]. Moreover, GO holds promise in environmental remediation for water purification and heavy metal removal [12].

Reduced graphene oxide (rGO) is produced through various reduction methods to overcome the insulating behaviour of GO, including chemical, thermal, and photothermal reduction to remove the oxygen functional groups [6,13,14]. The reduction process of GO significantly alters its structural, mechanical, and electrical properties. The removal of oxygen-containing groups and the restoration of the sp^2^ carbon structure lead to enhanced electrical conductivity (up to 6300 S cm^−1^), increased mechanical strength (Young’s modulus of ~1.0 TPa and breaking strength of ~130 GPa), high thermal stability, and high mobility (320 cm^2^ V^−1^ s^−1^), comparable to pristine graphene [6,15,16,17]. However, the dispersibility of rGO decreases due to its increased hydrophobicity [6,15,16,17].

Uniform dispersion and strong interfacial bonding between the polymer matrix and fillers remain challenging to achieve. The lack of compatibility between the components results in a significant difference in surface tension, leading to weak bonding at the interface and ultimately resulting in poor end-product properties [18]. Compatibilisers can be added to the blend to enhance the compatibility between PC/ABS and graphene-based fillers [19,20,21,22,23,24]. Compatibilisers are molecules that can be oriented at the interface between the two polymer phases, which decreases the strain at the interface and enhances compatibility [18].

The two main types of compatibilisers are known as non-reactive and reactive [25,26]. Non-reactive compatibilisers are the most common type and consist of segments or blocks with specific interaction or miscibility with one or both components [25,26]. The efficiency of non-reactive compatibilisers depends on the respective blocks and the molecular weight of the compatibiliser [26]. Reactive compatibilisers are formed at the interface between the two immiscible polymers [25,26]. These compatibilisers can be created via different processes, including trans-reaction; reactive production of graft, block, or weakly crosslinked copolymers; formation of ionically bound structures; and mechano-chemical blending [23,26,27,28]. The addition of a compatibiliser can significantly improve the properties of polymer blends. Compatibilisers can stabilise the morphology of the blend, resist phase separation, and improve mechanical properties by reducing interfacial tension and increasing interfacial adhesion [25,26].

Styrene-ethylene-butylene-styrene grafted with maleic anhydride (SEBS-g-MAH) is a new material that has recently attracted considerable interest because of its distinct characteristics and prospective uses in other fields. SEBS-g-MAH is a block copolymer composed of three distinct segments: styrene (S), ethylene-butylene (EB), and styrene (S) blocks [28]. The EB midblock provides elastomeric properties, while the styrene end blocks enhance the material’s processibility and compatibility with other polymers [29]. Introducing ionic functional groups such as maleic anhydride (MA) grafted onto the styrene blocks can impart hydrophilic and amphiphilic characteristics to the polymer blends [30,31,32,33]. This unique structure enables SEBS-g-MAH to exhibit a combination of elastomeric, thermoplastic, and interfacial properties, making it a versatile material for various applications [34].

This study investigates the formulation and characterisation of PC/ABS blends reinforced with GO and rGO, incorporating SEBS-g-MAH as a compatibiliser (PAGOS and PArGOS, respectively). The aim is to explore the effects of additions that synergistically enhance the mechanical, thermal, and rheological properties of the composites. By addressing the challenges associated with polymer immiscibility and filler dispersion, this study aims to contribute to developing high-performance polymer composites with potential applications in medical devices, automotive components, and electrical industries. The literature has not reported work concerning the mechanical, thermal, and rheological properties of PAGOS and PArGOS.

## 2. Materials and Methods

### 2.1. Materials

Polycarbonate/Acrylonitrile-Butadiene-Styrene (PC/ABS) commercial blends (Bayblend T85 XF) were supplied by Covestro AG (Leverkusen, Germany). This blend has a melt volume-flow rate of 19 cm^3^/10 min, melting temperature of 260–260 °C, and density of 1140 kg/m^3^. Graphene oxide (GO) and reduced graphene oxide (rGO) (CAS no. 7782-42-5 [Graphite]) were obtained from BT Corp Generiques Nano PVT. Ltd. (Bengaluru, India). GO consists of a 10–20 nm thin sheet with <10 µm lateral dimension, purity of 99%, and bulk density of 0.35 g/mL. rGO is a very light powder with a bulk density of 0.49 g/mL and an electrical conductivity of >107 Siemens per meter (along the *X* and *Y* axes). SEBS-g-MAH, under the trade name of Kraton FG 1901X, was supplied by Shell Chemical Co. (The Hague, The Netherlands). The formulations of the samples are stated in Table 1.

### 2.2. Methods

#### 2.2.1. Fabrication of Samples

**Figure 1:**
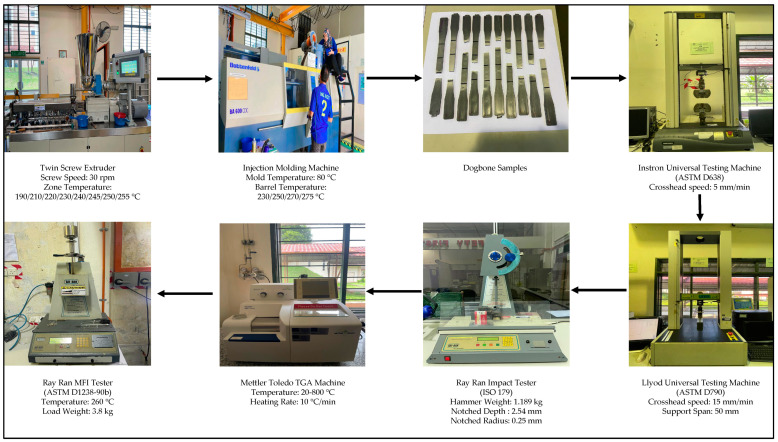
The schematic diagram of the production and analysis of the PAGO and PArGOS composites through melt blending.

Compounded materials were fabricated in a twin-screw extruder, with the recommended processing temperature for the barrel zone temperature steadily increasing as follows: 190/210/220/230/240/245/250/255 °C. The extrudates were then injection-moulded into standard tensile, flexural, and Charpy impact samples using an injection moulding machine. The moulding temperature was fixed at 80 °C, with the recommended processing temperature for the barrel zone steadily increasing as follows: 230/250/270/275 °C. The schematic diagram of the fabrication of samples are shown in Figure 1.

#### 2.2.2. Mechanical Properties Analysis

##### Tensile Testing

Mechanical property tests were performed on the composites. Each recipe was tested five times, and the average value was reported. The samples were tested for tensile strength following ASTM D638 utilising an Instron Universal Testing Machine at a speed of 5 mm/min [35]. The test specimen was kept at 23 °C for 48 h. The test was placed at room temperature.

##### Flexural Testing

According to ASTM D790, a three-point bending flexural test was performed using the Lloyd Universal Testing Machine at a speed of 15 mm/min [36]. The flexural strength and modulus were averaged from a minimum of five reported values.

##### Impact Testing

The notched Charpy impact test was conducted using a Ray-Ran Pendulum Impact Tester System (Ray-Ran Test Equipment Ltd., Warwickshire, UK) with a hammer weight of 1.189 kg, following ISO 179 [37]. Following the composite fabrication process, the notches, which had a depth of 2.54 mm and a radius of 0.25 mm, were machined. Each given value was obtained by testing a minimum of seven impact specimens, and the results were averaged.

#### 2.2.3. Thermal Properties Analysis

The thermal properties of the composites were assessed. The composites were subjected to thermal examination using a thermogravimetric analyser (TGA) (model 851e) (Mettler Toledo, OH, USA) under nitrogen combustion conditions. Prior to the test, the samples underwent a drying process for 2 h at a temperature of 100 °C to mitigate the influence of moisture. To examine the thermal deterioration characteristics, the temperature was systematically altered from 30 °C to 800 °C with a heating rate of 10 °C per minute in an environment of nitrogen.

#### 2.2.4. FTIR Analysis

The composites were tested with FTIR to investigate the functional groups’ presence in the samples. Nicolet i10s FTIR with attenuated total reflection (FTIR-ATR) was used to conduct the infrared spectroscopy investigations. Each spectrum was the result of 16 scans conducted at a spectral range from 4000 to 500 cm^−1^ with a resolution of 8 cm^−1^ using diamond ATR crystal. In the absorbance mode, the results were recorded in compliance with ASTM E168 [38].

#### 2.2.5. Rheological Properties Analysis

The melt flow index (MFI) of the blends was measured using the ASTM D1238-90b standard at a temperature of 260 °C and a load of 3.8 kg [39]. The apparatus utilised was a Ray-Ran (Model 5MPCA) melt flow index testing machine. Around 12 g of the sample was placed inside the barrel and heated to 260 °C. The sample was then allowed to melt and reach a state of thermal equilibrium for 4 min. The load was exerted on the molten substance, causing the material to be forced out through the die. The extrudates were sliced at consistent time intervals, typically every 20 s. The extrudates that were cut off were measured in terms of weight and then converted into units of grams per minute. The mean value of 3 extrudates was calculated for the MFI measurement.

## 3. Results and Discussion

### 3.1. FTIR

**Figure 2:**
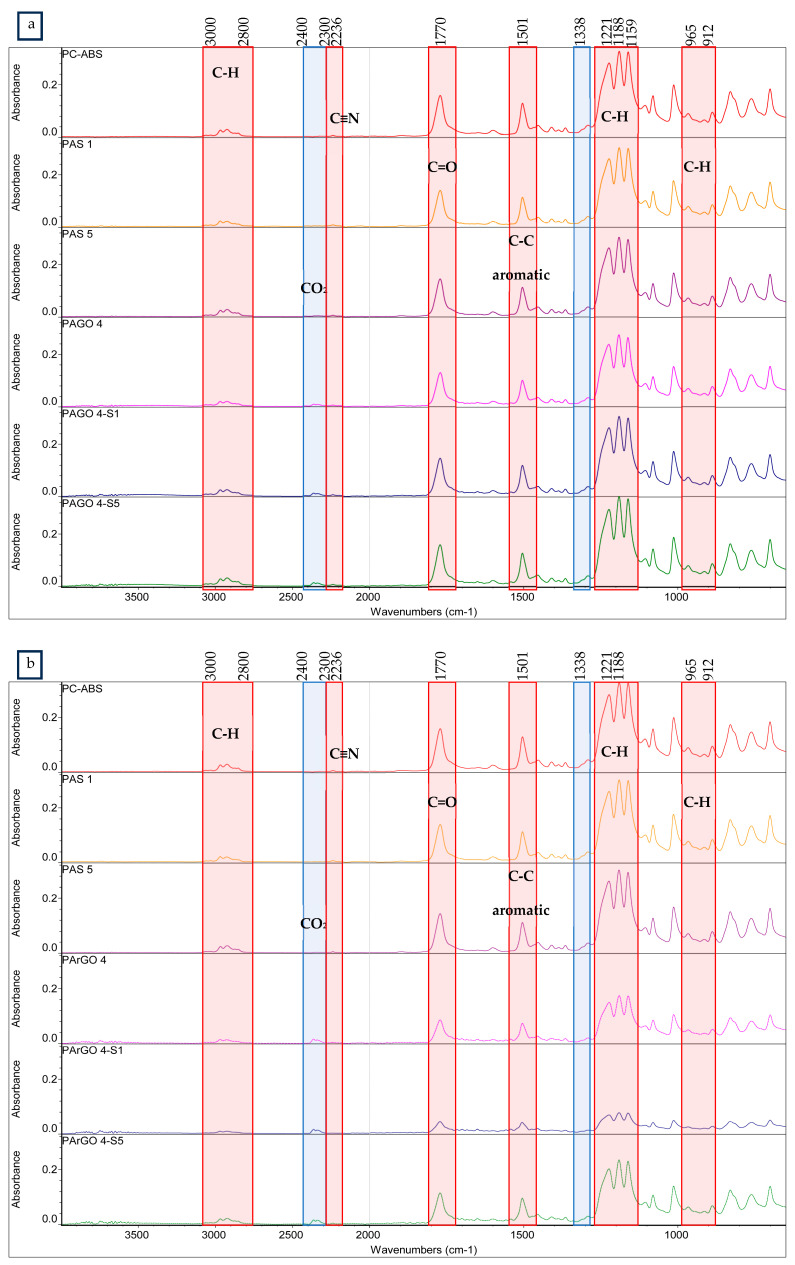

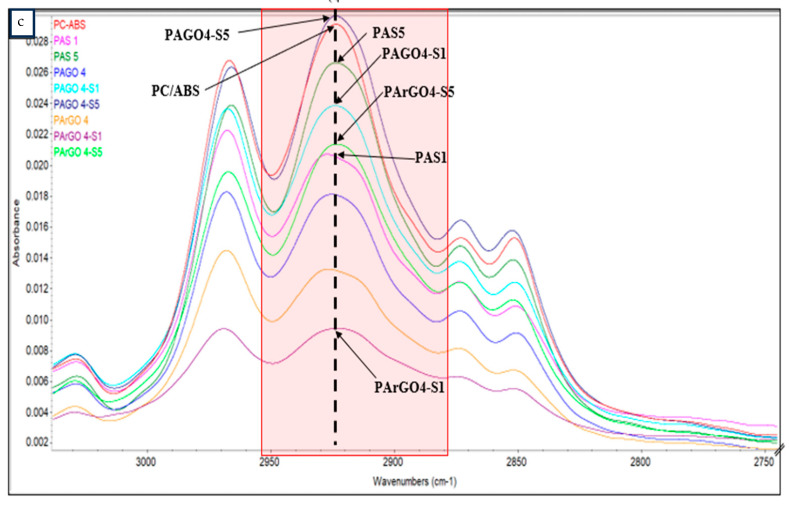
FTIR spectra of PC/ABS and PC/ABS composites at different SEBS-g-MAH loadings: (**a**) 4000–500 cm^−1^ for PAGO composites; (**b**) 4000–500 cm^−1^ for PArGO composites; and (**c**) 3000–2800 cm^−1^.

The FTIR was investigated to confirm the incorporation of SEBS-g-MAH into the composites. Figure 2 shows the FTIR spectra of PC/ABS composites with the presence of SEBS-g-MAH. The pristine PC/ABS spectrum exhibits deformation of the C–H bond of hydrogen atoms attached to the alkenic bond of 1,4-butadiene and 1,2-butadiene of ABS components at 965 cm^−1^ and 912 cm^−1^, respectively. The absorption of acrylonitrile in ABS can be seen at 2236 cm^−1^, indicating the presence of a nitrile stretching (C≡N) peak. Triple PC bands can be seen in all formulations at 1159 cm^−1^, 1188 cm^−1^, and 1221 cm^−1^, corresponding to C–O–C stretching, characterising the presence of ester bonds in PC/ABS. The absorption of the carbonyl (C=O) stretching peak of PC can be seen at 1770 cm^−1^. The spectra exhibit the methyl (C–H) stretching peaks at 3000 cm^−1^–2800 cm^−1^, presumably the aliphatic stretching and absorption of the methyl group of PC/ABS for all formulations.

No significant change in peak positions was observed in the PAGO and PArGO composites compared to pristine PC/ABS, as shown in Figure 2, suggesting that the incorporation of GO and rGO did not result in a drastic transformation in the chemical structure of the PC/ABS matrix. However, the incorporation of GO and rGO into the PC/ABS composites shows an increment in oxygen-containing functional groups at 1850 cm^−1^–1650 cm^−1^. The increment of visible peaks at 1570 cm^−1^–1530 cm^−1^ indicates the presence of GO and rGO carbon skeletons (C=C) due to thermal reduction, and the peak at 1637 cm^−1^ disappeared due to mechanical blending. A weak CO_2_ adsorption peak in range of 2400 cm^−1^–2300 cm^−1^ shows that GO and rGO were successfully incorporated into the composite.

From the FTIR spectra of the prepared composites, as shown in Figure 2, a slight decrease in C–H bands in the range of 3000 cm^−1^–2800 cm^−1^ resulted from incorporating 1 phr and 5 phr of SEBS-g-MAH into the PC/ABS composites, as reported by Rodrigues et al. [40]. The peak intensity decrease resulted from the formation of an anhydride group from the SEBS-g-MAH functional group interacting with hydroxyl in PC/ABS. Rodrigues et al. [40] also reported that grafting of the MAH group can occur via loss of vinylic hydrogen from the polybutadiene fraction or interaction with the C=C bond [23]. Thus, the grafting degree can be estimated by analysing the C≡N bond.

The IR spectra also showed a higher peak intensity at 2400 cm^−1^–2300 cm^−1^ with the addition of 1 and 5 phr of SEBS-g-MAH, which indicates an interaction between the MAH functional group and the hydroxyl and carboxyl groups of GO and rGO in PC/ABS.

**Figure 3:**
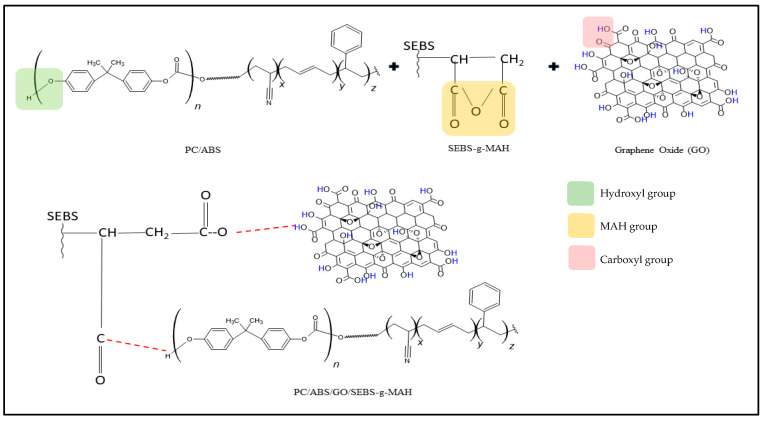
The possible interaction between the MAH group of SEBS-g-MAH with PC/ABS and GO.

The compatibilising effect was due to the interaction of SEBS chains in SEBS-g-MAH, which are compatible with ABS. The grafted maleic anhydride (C(=O)OC=O) in SEBS-g-MAH can react with the carboxyl (–COOH) and hydroxyl groups (O–H) of GO and rGO and the polybutadiene backbone chemical structure, leading to a coupling effect [40,41], as proposed in Figure 3. The terminal –OH group of PC is expected to react with MAH grafted on SEBS, as mentioned by Rodrigues et al. [40], who also proposed that the reaction will establish a chemical connection on the interphase of the elastomeric and PC phases without disrupting the core structure of SEBS or ABS.

### 3.2. Rheological Analysis

The addition of SEBS-g-MAH into the composites synergistically improved the processability and reduced the viscosity of the composites. Table 2 shows that the incorporation compatibiliser increased the MFI of the PC/ABS blends from 13.27 g/10 min to 17.79 g/10 min with 1 phr of SEBS-g-MAH. Further addition of compatibiliser into the PC/ABS blends slightly decreased the MFI to 17.07 g/10 min. This can be due to a better dispersibility of ABS in the PC matrix and better interfacial adhesion between PC and ABS [42,43,44].

The incorporation of compatibiliser in the PAGO composites synergistically improved the flowability from 12.68 g/10 min to 19.29 g/10 min and 19.31 g/10 min for 1 phr and 5 phr, respectively. SEBS-g-MAH also significantly enhanced the flowability from 11.14 g/10 min to 16.63 g/10 min and 17.98 g/10 min when added into PArGO at 1 and 5 phr, respectively. This is due to the chemical interaction of the maleic anhydride group with the oxygen functional groups of the PAGO and PArGO composites. Due to these interactions, the interfacial interaction between the matrix and fillers improved and increased the mobility of the polymer chain, hence leading to a higher MFI, which is reflected in the enhancement of the thermal properties of the composites. These results differ from those of Sousa Filho et al. [45], who reported that the incorporation of SEBS in PS/ABS significantly reduced the MFI, indicating more interaction between the components. However, they are broadly consistent with earlier studies on PC/ABS/SEBS-g-MA [31], PP/SEBS [46], and PET/HDPE/SEBS-g-MA [33].

The observed rheological enhancements have significant implications for the processing and application of these composites. The improved MFI implies that these materials can be processed at lower temperatures and pressures, saving energy and reducing production costs. Additionally, the enhanced flowability facilitates the fabrication of complex shapes and thin-walled components, broadening the application scope of these composites in the automotive, aerospace, and consumer electronics industries.

### 3.3. Mechanical Analysis

For this study, the blends were produced using the melt blending method in a twin-screw extruder, and the samples were produced using injection moulding according to ASTM standards. The composites were produced to evaluate the potential of SEBS-g-MAH as a compatibiliser between the matrix, namely PC/ABS, with the GO and rGO fillers, focusing on the mechanical, thermal, and physical characterisation. The mechanical properties reported are flexural strength (FS), flexural modulus (FM), tensile strength (TS), Young’s modulus (YM), elongation at break (EB), unnotched impact strength (IS_UN_), and notched impact strength (IS_N_). To enhance the readability and streamline the section, PC/ABS/GO/SEBS-g-MAH and PC/ABS/rGO/SEBS-g-MAH will be addressed as PAGOS and PArGOS composites, respectively.

The effect of different loadings of SEBS-g-MAH on the PC/ABS, PAGO, and PArGO systems on TS can be seen in Table 3. The trend shows an increment in TS at 1 phr before the further addition of SEBS-g-MAH at 5 phr deteriorated the composites’ TS values. The value of pristine PC/ABS is 38.84 MPa, and the TS values slightly increased to 39.38 MPa and then decreased to 37.80 MPa with the addition of 1 phr and 5 phr, respectively. This shows that SEBS-g-MAH is compatible with PC/ABS and forms a miscible system that can support localised deformation by the rubber phase in the matrix.

The addition of 4 phr of GO and rGO into the PC/ABS composites increased the strength by 3.4% and 9%, respectively. The homogenous distribution of filler throughout the matrix enhanced the strength of the matrix fibre interaction, which prevented the matrix from deforming drastically. The tensile properties show that the matrix with rGO particles has a higher tensile strength than the GO-filled composites. These results are in accordance with a previous study comparing GO and rGO; the study suggests that rGO promotes better properties compared to GO in PET/PBT/G composites [21]. The higher surface-to-volume ratio of rGO compared to GO provides more surface area for the interfacial bonding between the matrix and the filler [47]. Although GO is expected to have better properties due to its higher number of oxygen-containing groups, the planar geometry and higher surface area of rGO contribute to the better mechanical properties of the PC/ABS composites [47].

The incorporation of SEBS-g-MAH into the PAGO and PArGO systems showed a comparable TS at 1 phr and a decrease with 5 phr of compatibiliser in both systems. Adding 1 phr of SEBS-g-MAH into PAGO4.0 showed an insignificant reduction (*p* > 0.05) compared to uncompatibilised PAGO. A significant reduction (*p* < 0.05) could be seen with the addition of 5 phr of compatibiliser into the PAGO system, decreasing from 40.17 MPa to 36.60 MPa. A similar trend could be seen with the addition of the compatibiliser into the PArGO system, where the addition of SEBS-g-MAH showed an insignificant reduction at lower loadings and a significant reduction at higher amounts of SEBS-g-MAH loading compared to the compatibilised and uncompatibilised composites. This concurs with the findings by Abedin et al. [41] on the study of ABS/Talc/GO with SEBS-g-MAH, which reported insignificant enhancement of TS properties with the addition of compatibiliser into the composites. This shows that the addition of SEBS-g-MAH has a counteractive effect on the TS properties of the ternary systems of PAGO and PArGO composites.

A similar trend could be seen for YM in Table 3. The fillers showed significant improvement compared to pristine PC/ABS, as the YM increased to 1092 MPa and 1095 MPa for PAGO and PArGO, respectively. This study shows that the stiffness of the PAGO and PArGO composites increases as the filler increases. SEBS-g-MAH promotes the interfacial interaction between the PC and ABS phases, which leads to an improvement in tensile properties. Pristine PC/ABS has a value of 1041 MPa, and the addition of the compatibiliser increased the value from 1041 MPa to 1049 MPa and 1062 MPa with the addition of 1 phr and 5 phr, respectively. This is in accordance with a previous report by Debbah et al. [31] on the study of the PC/ABS ratio with SEBS-g-MAH, where the addition of the compatibiliser enhanced the dispersion of ABS in the PC matrix and the interfacial adhesion between PC and ABS [31]. The addition of the compatibiliser into PAGO and PArGO at all loading amounts significantly reduced the composites’ stiffness compared to the uncompatibilised composites. The addition of more than 1 phr of SEBS-g-MAH into the systems drastically reduced the stiffness of the composites. The addition of 5 phr of SEBS-g-MAH further reduced the stiffness from 1092 MPa to 1028 MPa and 1095 MPa to 1068 MPa for the PAGO and PArGO composites, respectively. The decrease in YM and TS could be explained by the elastomeric nature of the impact modifier as a toughening agent [48,49]. It is worth noting that the YM of the PArGOS system is higher than that of the PAGOS system at all loading amounts.

The EB of the PAGO and PArGO composites decreased with the addition of fillers, as illustrated in Table 3. For instance, the elongation at break of pristine PC/ABS decreased from 29% to 13% and 10% for the composite with 4 phr of GO and rGO, respectively. This is probably related to the higher surface-to-volume ratio of rGO compared to GO, as mentioned in the study conducted by Tayebi et al. [47] on incorporating GO and rGO into LDPE/EVA nanocomposites. The results concur well with a previous study, where the higher surface area available for bonding results in higher composite stiffness and decreased elongation at break. The incorporation of SEBS-g-MAH into the PC/ABS blends and the PAGO and PArGO composites showed a synergistic effect in increasing the elasticity of the composites. The trend showed a higher amount of SEBS-g-MAH in the system, further improving the elongation at break. The EB of PC/ABS blends increased from 29% to 51% with 1 phr of compatibiliser and decreased to 45% with further addition of SEBS-g-MAH. The presence of GO and rGO fillers in the PC/ABS blends drastically reduced the elasticity of the composites. A synergistic effect could be seen with the compatibiliser added to the PAGO composites, with an increment of up to 17% for 1 phr and 33% for 5 phr of SEBS-g-MAH. A similar trend could be seen with the addition of SEBS-g-MAH to the PArGO composites, where the EB increased by 8% and 73% when adding 1 phr and 5 phr of SEBS-g-MAH, respectively. As mentioned by Chow et al. [32] in their study of PLA/HNT/SEBS-g-MAH, the compatibiliser can induce an energy dissipation mechanism in the composites and elongate to a higher extent [32]. This confirms that SEBS-g-MAH contributes to synergistic compatibilisation, as the EB improved while the stiffness was reduced.

The effect of SEBS-g-MAH as a compatibiliser in PC/ABS is demonstrated in terms of flexural strength and modulus, as shown in Table 3. From the results, it can be seen that the addition of fillers significantly enhanced the FS and FM of the composites compared to pristine PC/ABS. The FS increased up to 7% and 10%, and the modulus also increased up to 8% and 15% for the PAGO and PArGO composites, respectively. The results show improvement as the compatibiliser is introduced into the system. For instance, adding 1 phr and 5 phr of SEBS-g-MAH into PC/ABS blends showed only an insignificant improvement from 29.55 MPa to 29.77 MPa and 30.44 MPa compared to the pristine PC/ABS. A similar trend with TS shows that the addition of 1 phr of the compatibiliser has comparable values with the uncompatibilised composites, and the properties deteriorate with higher loadings. When the amount of loading of SEBS-g-MAH was 1 phr, the FS was 31.97 MPa and the FM was 460 MPa for the PAGO composites, which further deteriorated to 30.82 MPa and 440 MPa, respectively. In the PArGO composites, the FS was 32.04 MPa and the FM was 464 MPa with the addition of 1 phr, and they further decreased to 31.48 MPa and 455 MPa with the addition of 5 phr of SEBS-g-MAH. This might be due to the high viscosity of the system and the agglomeration of compatibilisers in the systems. The results are in concordance with the study of SEBS-g-GO/Epoxy, which shows that a higher content of SEBS-g-GO in epoxy deteriorates the overall mechanical properties [23]. This shows that the compatibiliser has a counteractive effect on the FS and FM of the PAGO and PArGO composites.

The impact strength of PC/ABS-based composites was evaluated to study the material’s toughness and resistance to shock loading. Table 3 shows the notched and unnotched Charpy impact strengths of the PC/ABS blends, PAGO, and PArGO with different loadings of SEBS-g-MAH. The addition of fillers drastically decreased the impact strength of the composites. The PArGO composite showed lower impact strength compared to PAGO. In order to improve the impact properties, an impact modifier was introduced. The unnotched impact strength clearly showed synergistic reinforcement with the addition of SEBS-g-MAH as an impact modifier. The IS_UN_ showed an increment from 54.11 kJ/m^2^ to 75.14 kJ/m^2^ and 86.69 kJ/m^2^ with the addition of 1 phr and 5 phr of compatibiliser. The toughening agent synergistically improved the IS_UN_ PAGO composites up to 64% and 75% as the amount of loading increased. There was a slight reduction in IS_UN_ with the addition of 1 phr of SEBS-g-MAH from 52.54 kJ/m^2^ to 51.18 kJ/m^2^, which might be due to the void that was present within the composites [45]. A higher loading of SEBS-g-MAH synergistically enhanced the impact strength by up to 32% for the PArGO composites. The increment could be due to the toughening effect of the impact modifier within the ternary PAGO and PArGO composites, which caused effective load transfer and led to higher cross-linking [15]. The impact strength for notched Charpy impact strength (IS_N_) showed an improvement in IS with the addition of SEBS-g-MAH as an impact modifier into the PAGO composites. The higher the SEBS-g-MAH content in the system, the higher the IS _N_. The IS_N_ for the PAGO composites increased from 9.70 kJ/m^2^ to 10.50 kJ/m^2^ and 11.47 kJ/m^2^ for 1 phr and 5 phr, respectively. However, the addition of SEBS-g-MAH into the PArGO systems showed an insignificant reduction in the IS_N_. The enhancement of impact strength in the PAGO composites could be due to the large surface area of GO and the high oxygen content in the system compared to rGO.

It is noteworthy to observe that SEBS-g-MAH has higher synergistic reinforcement with PAGO compared to PArGO composites. This can be confirmed by EB, IS_N_, and IS_UN_, which show much higher enhancement in the PAGO composites. Thus, it can be concluded that SEBS-g-MAH acts as an impact modifier in the PAGO composites by enhancing the ductility and toughness of the composites.

**Figure 4:**
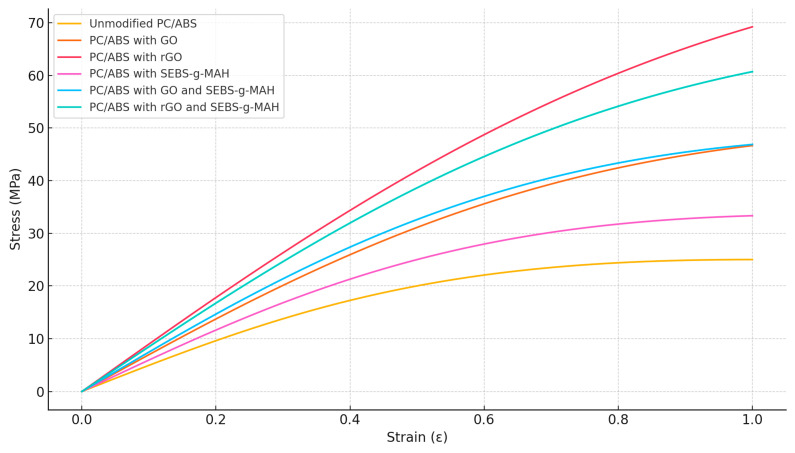
Representative stress–strain curves for PC/ABS with different additives.

The incorporation of graphene oxide (GO), reduced graphene oxide (rGO), and SEBS-g-MAH compatibiliser to PC/ABS thermoplastics dramatically modified the mechanical properties, as seen by the stress–strain curves provided in Figure 4. The changes are needed to enhance certain properties, such as strength, stiffness, and toughness, which are crucial for a variety of engineering applications. The unmodified PC/ABS blend shows typical ductile behaviour with a distinct yield point, followed by strain hardening and necking. Adding GO increased stiffness and yield strength, as indicated by the higher initial slope and elevated yield point. However, this also reduced ductility, making the material more brittle. rGO further enhanced mechanical properties, resulting in a higher modulus and yield strength compared to GO. Despite this, ductility decreased even more, which could limit applications requiring significant elongation. The SEBS-g-MAH compatibiliser improves interfacial adhesion between the polymer matrix and the fillers, leading to balanced improvements in strength and toughness. The stress–strain curve shows increased modulus and yield strength, with significant elongation at break, indicating enhanced toughness. Combining GO or rGO with SEBS-g-MAH produced a synergistic effect, resulting in composites with a high modulus, yield strength, and improved toughness. These materials exhibit high initial slopes and yield points, with better elongation at break compared to those without the compatibiliser, making them suitable for applications requiring both high strength and ductility.

### 3.4. Thermal Analysis

**Figure 5:**
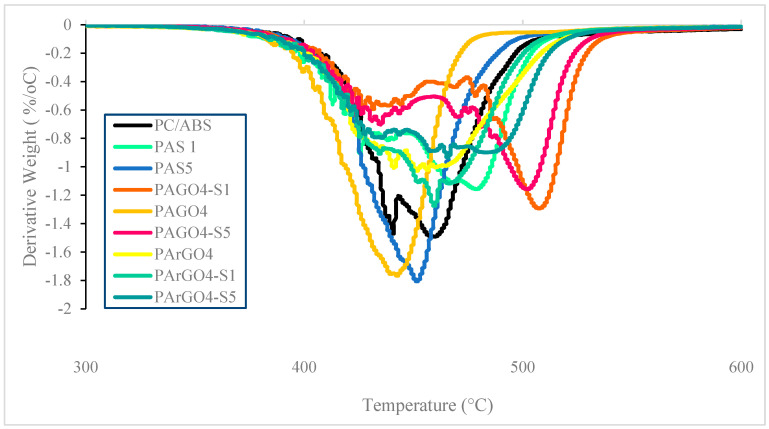
DTG curve for PC/ABS and PC/ABS-based composites at different SEBS-g-MAH loadings.

The TGA thermal degradation and differential thermogravimetry (DTG) of PC/ABS, PAGO, and PArGO with different amounts of SEBS-g-MAH loading and the values of the weight loss at different temperatures are summarised in Table 4 and illustrated in Figure 5. The data analysis clearly shows that the PAGO and PArGO composites incorporating compatibiliser have higher thermal stability than the pristine PC/ABS blend. T_10%_, T_50%_, and T_80%_ are the decomposition temperatures at 10, 50, and 80% of weight loss, respectively.

Figure 5 clearly shows that PC/ABS underwent two-step degradation, with the first decomposition attributed to ABS and the second degradation attributed to PC [39,42,50]. The main weight loss occurred in the region of 400 °C to 540 °C for all samples due to the decomposition of the organic components of the composites. All the composites show higher thermal stability compared to pristine PC/ABS. The incorporation of SEBS-g-MAH into PC/ABS showed an improvement in thermal stability, as the degradation temperature shifted to a higher temperature from 488 °C to 497 °C at 1 phr, but then decreased to 481 °C at 5 phr at 80% weight loss. The residual weight at 800 °C also seemed to increase from 6.25% to 11.93% and 9.82% at 1 phr and 5 phr, respectively, representing the composite’s compatibiliser amount. This concurs well with previous studies, which suggest enhanced interfacial interaction between the MAH group of SEBS-g-MAH and the terminal hydroxyl group of the composites [31,51,52,53,54,55,56].

The addition of SEBS-g-MAH clearly showed a synergistic effect with the GO filler as the thermal stability for the PAGOS composites at 1 phr and 5 phr increased. From the DTG curve, it can be observed that the composites underwent two-step degradation compared to one-step degradation without the presence of a compatibiliser. At 1 phr, the thermal stability of PAGOS increased by 10% and 17% compared to pristine PC/ABS and PAGO without compatibiliser, respectively. However, the thermal stability decreased when a higher loading of 5 phr was added. The enhancement in thermal stability is attributed to better cross-linking between PC/ABS and GO with the presence of a compatibiliser [15]. This TGA trend concurs well with [23], which indicates that SEBS-g-GO contributes to the barrier effect, which hinders volatilisation and enhances thermal stability. The char residue increased from 4.80% to 14.66% and 13.63% at 1 phr and 5 phr, respectively. Higher char residue at 800 °C indicates that the presence of SEBS-g-MAH improved the thermal oxidative resistance, resulting in increased char residue [23].

The addition of SEBS-g-MAH in the PArGO composites showed synergistic effects, as the most significant rate of change, known as the inflection point, shifted to a higher temperature compared to uncompatibilised PArGO. The inflection point for the addition of 1 phr was 458 °C, and for 5 phr it was 464 °C. The addition of compatibiliser into PArGO resulted in only one-step degradation. The char residue for PArGOS at 1 phr and 5 phr decreased with higher compatibiliser loading. This proves that SEBS-g-MAH synergistically improved the thermal stability of the composites.

## 4. Conclusions

The embedment and incorporation of GO and rGO have been successfully produced using the melt blending method without the involvement of any chemical alteration during the fabrication. The synthesis of PC/ABS composites filled with GO, rGO, and SEBS-g-MAH was proven by FTIR peak shifts and changes in intensity, as discussed previously in the FTIR sections. Although the FTIR of the PC/ABS blends and PAGO, PArGO, PAGOS, and PArGOS retain the original spectra of PC/ABS blends, the changes in peak intensities and peak shift show the possibility of bond formations and stacking of functional groups between the matrix and the fillers.

The shifting to higher temperatures in TGA for PAGO, PArGO, PAGOS, and PArGOS shows that the fillers and the compatibiliser are embedded in the matrix, proving that the composites have high thermal stability, which also reflects enhanced mechanical properties, as discussed earlier.

From the results, it can be concluded that the incorporation of fillers does impact the ductility of the composite but offers numerous advantages, such as high stiffness, strength, and thermal stability, compared to pristine PC/ABS. The optimum amount of fillers with enhanced mechanical and thermal properties is 4 phr. The incorporation of fillers increased the thermal stability by up to 5% for PArGO4.0, with the enhancement of tensile strength (TS), flexural strength (FS), and flexural modulus (FM) by up to 9%, 10%, and 15%, respectively. The enhancement of the PAGO composites was due to a 5% increase in the stiffness of the composites. The addition of SEBS-g-MAH into the composites shows an influence on thermal stability, with the compatibiliser shifting the thermal degradation temperature up to 521 °C for PAGO4.0-S5 without significantly influencing the enhancement of mechanical properties.

## Figures and Tables

**Table 1:** Formulations of samples.

No.	Sample	PC/ABS (phr)	GO (phr)	rGO (phr)	SEBS-g-MAH (phr)
1	PC/ABS	100.0	-	-	-
2	PAS1		-	-	1
3	PAS5		-	-	5
4	PAGO4.0	100.0	4.0	-	-
5	PArGO4.0	100.0	-	4.0	-
6	PAGO4.0-S1	100.0	4.0	-	1
7	PAGO4.0-S5	100.0	4.0	-	5
8	PArGO4.0-S1	100.0	-	4.0	1
9	PArGO4.0-S5	100.0	-	4.0	5

**Table 2:** Melt flow index (MFI) of PC/ABS composites.

Samples	PC/ABS	PAS-1	PAS-5	PAGO4.0	PAGO4.0-S1	PAGO4.0-S5	PArGO4.0	PArGO4.0-S1	PArGO4.0-S5
MFI (g/10 min) 260 °C, 3.8 kg	13.27	17.79	17.07	12.68	19.29	19.31	11.14	16.63	17.98

**Table 3:** Mechanical Properties of PC/ABS composites.

Sample	TS (MPa)	YM (MPa)	E_B_ (%)	FS (MPa)	FM (MPa)	IS_N_ (kJ/m^2^)	IS_UN_ (kJ/m^2^)
PC/ABS	38.84 ± 1.2 ^c,d,e^	1041.60 ± 27.4 ^c,d^	29.09 ± 10.8 ^b^	29.55 ± 0.9 ^f^	421.52 ± 17.3 ^e^	38.68 ± 0.4 ^a^	54.11 ± 8.6 ^d,e^
PAS-1	39.38 ± 0.4 ^b,c,d^	1049.40 ± 20.9 ^b,c,d^	51.33 ± 9.0 ^a^	29.77 ± 0.1 ^e,f^	433.94 ± 10.0 ^d,e^	37.32 ± 0.3 ^a^	75.14 ± 4.6 ^b,c^
PAS-5	37.80 ± 1.3 ^d,e^	1062.40 ± 33.0 ^a,b,c,d^	45.83 ± 3.4 ^a^	30.44 ± 0.2 ^d,e^	423.74 ± 4.3 ^e^	36.91 ± 3.0 ^a^	86.69 ± 13.8 ^b^
PAGO-4.0	40.17 ± 1.1 ^a,b,c^	1092.40 ± 31.7 ^a,b^	13.14 ± 1.3 ^d^	31.57 ± 0.2 ^b,c^	456.24 ± 2.2 ^b,c^	9.70 ± 0.2 ^d^	48.75 ± 5.4 ^e^
PArGO-4.0	42.35 ± 1.1 ^a^	1095.00 ± 13.4 ^a^	9.97 ± 1.4 ^d^	32.39 ± 0.2 ^a^	485.28 ± 3.6 ^a^	5.47 ± 0.2 ^e^	52.54 ± 1.8 ^e^
PAGO4.0-S1	39.82 ± 0.5 ^b,c,d^	1067.60 ± 18.7 ^a,b,c,d^	15.40 ± 2.1 ^c,d^	31.97 ± 0.2 ^a,b^	460.11 ± 4.0 ^b^	10.50 ± 0.2 ^d^	79.97 ± 3.9 ^b,c^
PAGO4.0-S5	36.60 ± 0.5 ^e^	1028.20 ± 12.5 ^d^	17.51 ± 1.4 ^b,c,d^	30.82 ± 0.7 ^c,d^	440.71 ± 4.8 ^c,d^	11.47 ± 0.2 ^d^	85.28 ± 12.3 ^b,c^
PArGO4.0-S1	41.36 ± 0.7 ^a,b^	1074.80 ± 15.5 ^a,b,c^	10.84 ± 1.6 ^d^	32.04 ± 0.2 ^a,b^	464.16 ± 7.3 ^b^	4.66 ± 0.2 ^e^	51.18 ± 2.7 ^e^
PArGO4.0-S5	37.93 ± 2.1 ^c,d,e^	1068.80 ± 11.3 ^a,b,c,d^	17.28 ± 1.1 ^b,c,d^	31.48 ± 0.1 ^b,c^	455.36 ± 7.1 ^b,c^	4.61 ± 0.3 ^e^	69.57 ± 4.6 ^c,d^

Any results which do not share a letter are significantly different (*p* < 0.05).

**Table 4:** TGA data of PC/ABS, PAGOS, and PArGOS at different amounts of SEBS-g-MAH loading.

Sample	Degradation Temperature				Inflection Point (°C)	Char Residue at 800 °C (%)
	T_5%_	T_10%_	T_50%_	T_80%_		
PC/ABS	410	423	457	488	462	6.24
PAS1	400	415	466	497	478	11.93
PAS5	408	420	451	481	445	9.82
PAGO04.0	357	402	438	461	438	4.80
PAGO4.0-S1	403	420	497	538	502	14.66
PAGO4.0-S5	404	419	486	521	497	13.63
PArGO4.0	397	415	449	512	449	14.40
PArGO4.0-S1	400	415	461	502	458	14.17
PArGO4.0-S5	402	417	469	510	464	13.69

## Data Availability

The data presented in this study are available upon request from the corresponding author.

## References

[1] Hassan A., Yean Jwu W. Mechanical Properties of High Impact ABS/PC Blends-Effect of Blend Ratio. Proceedings of the Simposium Polimer Kebangsaan ke-V, Hotel Residence.

[2] Khun N.W., Liu E. (2013). Thermal, Mechanical and Tribological Properties of Polycarbonate/Acrylonitrile-Butadiene-Styrene Blends. J. Polym. Eng..

[3] Masud M., Khan K., Gupta R., Agarwal S., Khan M.M.K., Liang R.F., Gupta R.K., Agarwal S. (2005). Rheological and Mechanical Properties of ABS/PC Blends. Korea-Aust. Rheol. J..

[4] Dal Lago E., Cagnin E., Boaretti C., Roso M., Lorenzetti A., Modesti M. (2020). Influence of Different Carbon-Based Fillers on Electrical and Mechanical Properties of a PC/ABS Blend. Polymers.

[5] dos Anjos E.G.R., Braga N.F., Ribeiro B., Escanio C.A., Cardoso A.d.M., Marini J., Antonelli E., Passador F.R. (2022). Influence of Blending Protocol on the Mechanical, Rheological, and Electromagnetic Properties of PC/ABS/ABS-g-MAH Blend-Based MWCNT Nanocomposites. J. Appl. Polym. Sci..

[6] Razaq A., Bibi F., Zheng X., Papadakis R., Jafri S.H.M., Li H. (2022). Review on Graphene-, Graphene Oxide-, Reduced Graphene Oxide-Based Flexible Composites: From Fabrication to Applications. Materials.

[7] Morales-Zamudio L., Lozano T., Caballero-Briones F., Zamudio M.A.M., Angeles-San Martin M.E., de Lira-Gomez P., Martinez-Colunga G., Rodriguez-Gonzalez F., Neira G., Sanchez-Valdes S. (2021). Structure and Mechanical Properties of Graphene Oxide-Reinforced Polycarbonate. Mater. Chem. Phys..

[8] Smith A.T., LaChance A.M., Zeng S., Liu B., Sun L. (2019). Synthesis, Properties, and Applications of Graphene Oxide/Reduced Graphene Oxide and Their Nanocomposites. Nano Mater. Sci..

[9] Zhu Y., Murali S., Cai W., Li X., Suk J.W., Potts J.R., Ruoff R.S. (2010). Graphene and Graphene Oxide: Synthesis, Properties, and Applications. Adv. Mater..

[10] Joy A., Unnikrishnan G., Megha M., Haris M., Thomas J., Deepti A., Baby Chakrapani P.S., Kolanthai E., Muthuswamy S. (2023). A Novel Combination of Graphene Oxide/Palladium Integrated Polycaprolactone Nanocomposite for Biomedical Applications. Diam. Relat. Mater..

[11] Esmaeili E., Eslami-Arshaghi T., Hosseinzadeh S., Elahirad E., Jamalpoor Z., Hatamie S., Soleimani M. (2020). The Biomedical Potential of Cellulose Acetate/Polyurethane Nanofibrous Mats Containing Reduced Graphene Oxide/Silver Nanocomposites and Curcumin: Antimicrobial Performance and Cutaneous Wound Healing. Int. J. Biol. Macromol..

[12] Banerjee A.N. (2018). Graphene and Its Derivatives as Biomedical Materials: Future Prospects and Challenges. Interface Focus.

[13] Ibrahim A., Klopocinska A., Horvat K., Hamid Z.A. (2021). Graphene-Based Nanocomposites: Synthesis, Mechanical Properties, and Characterizations. Polymers.

[14] Wu X.H., Then Y.Y. (2021). Fabrication and Characterization of Superhydrophobic Graphene/Titanium Dioxide Nanoparticles Composite. Polymers.

[15] Aradhana R., Mohanty S., Nayak S.K. (2018). Comparison of Mechanical, Electrical and Thermal Properties in Graphene Oxide and Reduced Graphene Oxide Filled Epoxy Nanocomposite Adhesives. Polymer.

[16] Hidayah N.M.S., Liu W.W., Lai C.W., Noriman N.Z., Khe C.S., Hashim U., Lee H.C. (2017). Comparison on Graphite, Graphene Oxide and Reduced Graphene Oxide: Synthesis and Characterization. AIP Conf. Proc..

[17] Ray S.C. (2015). Application and Uses of Graphene Oxide and Reduced Graphene Oxide. Applications of Graphene and Graphene-Oxide Based Nanomaterials.

[18] Qiao Y., Fring L.D., Pallaka M.R., Simmons K.L. (2023). A Review of the Fabrication Methods and Mechanical Behavior of Continuous Thermoplastic Polymer Fiber–Thermoplastic Polymer Matrix Composites. Polym. Compos..

[19] Shi H., Shi D., Li C., Luan S., Yin J., Li R.K.Y. (2014). Preparation of Functionalized Graphene/SEBS-g-MAH Nanocomposites and Improvement of Its Electrical, Mechanical Properties. Mater. Lett..

[20] Rafiee M., Nitzsche F., Laliberte J., Hind S., Robitaille F., Labrosse M.R. (2019). Thermal Properties of Doubly Reinforced Fiberglass/Epoxy Composites with Graphene Nanoplatelets, Graphene Oxide and Reduced-Graphene Oxide. Compos. B Eng..

[21] Ucpinar Durmaz B., Atılgan M.G., Aytac A. (2022). A Comparative Study of Graphene Oxide or Chemically Reduced Graphene Oxide Filled Poly(Ethylene Terephthalate)/Poly(Butylene Terephthalate)/Graphene Nanocomposites. Iran. Polym. J..

[22] Inuwa I.M., Arjmandi R., Ibrahim A.N., Haafiz M.K.M., Wong S.L., Majeed K., Hassan A. (2016). Enhanced Mechanical and Thermal Properties of Hybrid Graphene Nanoplatelets/Multiwall Carbon Nanotubes Reinforced Polyethylene Terephthalate Nanocomposites. Fibers Polym..

[23] Wei L., Chen X., Hong K., Yuan Z., Wang L., Wang H., Qiao Z., Wang X., Li Z., Wang Z. (2019). Enhancement in Mechanical Properties of Epoxy Nanocomposites by Styrene-Ethylene-Butadiene-Styrene Grafted Graphene Oxide. Compos. Interfaces.

[24] Wang C., Ge H., Ma X., Liu Z., Wang T., Zhang J. (2018). Effect of Graphene Oxide Mixed Epoxy on Mechanical Properties of Carbon Fiber/Acrylonitrile-Butadiene-Styrene Composites. J. Nanosci. Nanotechnol..

[25] Ajitha A.R., Thomas S. (2019). Introduction: Polymer Blends, Thermodynamics, Miscibility, Phase Separation, and Compatibilization. Compatibilization of Polymer Blends: Micro and Nano Scale Phase Morphologies, Interphase Characterization, and Properties.

[26] Ajitha A.R., Mathew L.P., Thomas S. (2019). Compatibilization of Polymer Blends by Micro and Nanofillers. Compatibilization of Polymer Blends: Micro and Nano Scale Phase Morphologies, Interphase Characterization, and Properties.

[27] Olongal M., Raphael L.R., Raghavan P., Mohamed Nainar M.A., Athiyanathil S. (2021). Maleic Anhydride Grafted Acrylonitrile Butadiene Styrene (ABS)/Zinc Oxide Nanocomposite: An Anti-Microbial Material. J. Polym. Res..

[28] Zhang X.L., Wu H., Guo S.Y. (2015). The Molecular Structure of SEBS Grafted with Maleic Anhydride through Ultrasound Initiation. Chin. J. Polym. Sci. (Engl. Ed.).

[29] Cai H., Lu T., Jiang Y., Chen J., Xiao Y., Han B., Gao W., Ju J. (2023). Experimental and Computational Investigation on Performances of the Thermoplastic Elastomer SEBS/Poly(Lactic Acid) Blends. Mater. Today Commun..

[30] Zhu N., Gao X., Liang J., Wang Y., Hou R., Ni Z. (2022). Finely Modulated LDPE/PS Blends via Synergistic Compatibilization with SEBS-g-MAH and OMMT. Symmetry.

[31] Debbah I., Krache R., Aranburu N., Fernández M., Etxeberria A. (2018). Effect of SEBS-g-MAH Addition on the Mechanical, Rheological, and Morphological Properties of Polycarbonate/Acrylonitrile–Butadiene–Styrene Blends. J. Elastomers Plast..

[32] Chow W.S., Tham W.L., Poh B.T., Mohd Ishak Z.A. (2018). Mechanical and Thermal Oxidation Behavior of Poly(Lactic Acid)/Halloysite Nanotube Nanocomposites Containing N,N′-Ethylenebis(Stearamide) and SEBS-g-MA. J. Polym. Environ..

[33] Taghavi S.K., Shahrajabian H., Hosseini H.M. (2018). Detailed Comparison of Compatibilizers MAPE and SEBS-g-MA on the Mechanical/Thermal Properties, and Morphology in Ternary Blend of Recycled PET/HDPE/MAPE and Recycled PET/HDPE/SEBS-g-MA. J. Elastomers Plast..

[34] Song L., Cong F., Wang W., Ren J., Chi W., Yang B., Zhang Q., Li Y., Li X., Wang Y. (2023). The Effect of Functionalized SEBS on the Properties of PP/SEBS Blends. Polymers.

[35] (2022). Standard Test Method for Tensile Properties of Plastics.

[36] (2017). Standard Test Methods for Flexural Properties of Unreinforced and Reinforced Plastics and Electrical Insulating Materials.

[37] (2020). Plastics—Determination of Charpy Impact Properties—Part 2: Instrumented Impact Test.

[38] (2023). Standard Practices for General Techniques of Infrared Quantitative Analysis.

[39] (2017). Standard Test Method for Flow Rates of Thermoplastics by Extrusion Plastometer.

[40] Rodrigues P.V., Ramoa B., Torres A.R., Castro M.C.R., Machado A.V. (2023). Enhancing the Interface Behavior on Polycarbonate/Elastomeric Blends: Morphological, Structural, and Thermal Characterization. Polymers.

[41] Tejada-Oliveros R., Balart R., Ivorra-Martinez J., Gomez-Caturla J., Montanes N., Quiles-Carrillo L. (2021). Improvement of Impact Strength of Polylactide Blends with a Thermoplastic Elastomer Compatibilized with Biobased Maleinized Linseed Oil for Applications in Rigid Packaging. Molecules.

[42] Jamaludin N.A., Hassan A., Othman N., Jawaid M. (2015). Effects of Halloysite Nanotubes on Mechanical and Thermal Stability of Poly(Ethylene Terephthalate)/Polycarbonate Nanocomposites. Appl. Mech. Mater..

[43] Asyadi F., Jawaid M., Hassan A., Wahit M.U. (2013). Mechanical Properties of Mica-Filled Polycarbonate/Poly(Acrylonitrile-Butadiene-Styrene) Composites. Polym.-Plast. Technol. Eng..

[44] Pour R.H., Hassan A., Soheilmoghaddam M., Bidsorkhi H.C. (2016). Mechanical, Thermal, and Morphological Properties of Graphene Reinforced Polycarbonate/Acrylonitrile Butadiene Styrene Nanocomposites. Polym. Compos..

[45] de Sousa Filho V.A., de Albuquerque Filho M.A., de Alencar Lira M.C., Pedrosa T.C., da Fonseca L.S., Araújo S.S., Henrique M.A., da Silva Barbosa Ferreira E., Araújo E.M., Luna C.B.B. (2023). Efficiency Assessment of the SEBS, SEP, and SBS Copolymers in the Compatibilization of the PS/ABS Blend. J. Polym. Res..

[46] Matei E., Râpă M., Andras Á.A., Predescu A.M., Pantilimon C., Pica A., Predescu C. (2017). Recycled Polypropylene Improved with Thermoplastic Elastomers. Int. J. Polym. Sci..

[47] Tayebi M., Ramazani S.A.A., Hamed Mosavian M.T., Tayyebi A. (2015). LDPE/EVA/Graphene Nanocomposites with Enhanced Mechanical and Gas Permeability Properties. Polym. Adv. Technol..

[48] Tjong S.C., Meng Y.Z. (1999). Effect of Reactive Compatibilizers on the Mechanical Properties of Polycarbonate/Poly(Acrylonitrile-Butadiene-Styrene) Blends. Eur. Polym. J..

[49] Tjong S.C., Bao S.P., Liang G.D. (2005). Polypropylene/Montmorillonite Nanocomposites Toughened with SEBS-g-MA: Structure-Property Relationship. J. Polym. Sci. B Polym. Phys..

[50] Mazlan M.A.S., Zakaria Z., Ahmad Saidi M.A., Hassan A., Xin C.J. (2020). Effect of Graphene Oxide on Mechanical, Thermal and Physical Properties of Impact Modified Poly(Lactic Acid) Nanocomposites. PERINTIS eJournal.

[51] Li H., Zhao J., Liu S., Yuan Y. (2014). Polycarbonate–Acrylonitrile-Butadiene-Styrene Blends with Simultaneously Improved Compatibility and Flame Retardancy. RSC Adv..

[52] Hou S., Li Z., Zhang Y.J., Jiang P. (2021). Phosphorous-Phosphorous Synergistic Effect on Flame Retardancy, Mechanically Reinforce and Hydrolytic Resistance for PC/ABS Blends. Polym. Degrad. Stab..

[53] Qiao Z., Ma Y., Chen X., Chen M., Hong K., Li Z., Lu G., Wang Z. (2020). Mechanical and Piezo-resistive Properties of Functionalized Multi-walled Carbon Nanotubes/Styrene-ethylene-butadiene-styrene Composites. Polym. Compos..

[54] Jamaludin N.A., Inuwa I.M., Hassan A., Othman N., Jawaid M. (2015). Mechanical and Thermal Properties of SEBS-g-MA Compatibilized Halloysite Nanotubes Reinforced Polyethylene Terephthalate/Polycarbonate/Nanocomposites. J. Appl. Polym. Sci..

[55] Han W., Wu M., Rong J., Zhang S., Zhang X., Zhao T., Yu X., Naito K., Zhang Q. (2023). Effect of Functionalized Nanodiamond on Properties of Polylactic Acid Eco-Friendly Composite Films. Diam. Relat. Mater..

[56] Kusmono, Mohd Ishak Z.A., Chow W.S., Takeichi T. (2008). Rochmadi Influence of SEBS-g-MA on Morphology, Mechanical, and Thermal Properties of PA6/PP/Organoclay Nanocomposites. Eur. Polym. J..

